# The Role of Endoplasmic Reticulum Stress in Gastroesophageal Reflux Disease Symptoms and Treatment

**DOI:** 10.5152/tjg.2023.21282

**Published:** 2023-05-01

**Authors:** Elmas Kasap, Tahir Buran, Aysun Toraman Avcu, Pınar Solmaz Hasdemir, Erdal Balcan, Çağdaş Aktan, Mehmet Korkmaz

**Affiliations:** 1 Department of Gastroenterology, Manisa Celal Bayar University Faculty of Medicine, Manisa, Turkey; 2 Department of Medical Biology, Manisa Celal Bayar University Faculty of Medicine, Manisa, Turkey; 3 Division of Biology, Department of Molecular Biology, Manisa Celal Bayar University Faculty of Arts and Science, Manisa, Turkey; 4 Department of Medical Biology, University of Beykent Faculty of Medicine, İstanbul, Turkey; 5 Department of Nephrology, Manisa Celal Bayar University Faculty of Medicine, Manisa, Turkey; 6 Department of Obstetrics, Manisa Celal Bayar University Faculty of Medicine, Manisa, Turkey

**Keywords:** Endoplasmic reticulum stress, GERD, heartburn, proton pump inhibitors, regurgitation

## Abstract

**Background:**

Gastroesophageal reflux disease is a common condition worldwide. There is no curative treatment for gastroesophageal reflux disease. Endoplasmic reticulum stress leads to the activation of the unfolded protein response and has an important role in inflammation. The aim is to determine the role of endoplasmic reticulum stress in the follow-up of individuals with gastroesophageal reflux disease and the temporal changes of endoplasmic reticulum stress markers with treatment.

**Methods:**

Twenty-four subjects in total were recruited prospectively, of whom 15 had nonerosive reflux disease. Two biopsies from 2 cm above the esophagogastric junction, 2 biopsies from gastric antrum mucosa, and 2 biopsies from gastric corpus mucosa were taken. Simultaneously, 2 tubes of venous blood samples were drawn from each individual (1 tube for studying the genetic markers and 1 tube for analyzing the *CYP2C19* polymorphism).

**Results:**

The mean age was 42.3 ± 17.6 for women and 34.66 ± 11.2 for men. Pantoprazole, esomeprazole, rabeprazole, and lansoprazole preparations were used for treatment. There was no significant difference between tissue and blood samples for panel genes *ATF-6*, *XBP-1*, *DDIT-3*, *DNAJC-10*, and *EIF-2-AK* before treatment. There was a significant decrease in the level of ATF-6, *XBP-1*, *DNAJC-9*, *EIF-2-AK*, and *NF-2L-2* genes in blood after treatment. In the comparison of proton pump inhibitors, significant decreases in the expression of the *ATF-6*, *XBP-1*, and *DNAJC-9* mRNAs were detected in blood from individuals after treatment.

**Conclusion:**

Endoplasmic reticulum stress can be for evaluating the clinical improvement and the effectiveness of treatment in gastroesophageal reflux disease.

**Main Points**

- We examined the role of endoplasmic reticulum stress in the follow-up of individuals with gastroesophageal reflux disease and the temporal changes with treatment.
- The patients’ blood and tissue values were compared before the treatment and blood values, gender, *Helicobacter pylori*, esophagitis, *CYP2c19-2*, and proton pump inhibitors were compared before and after the treatment.
- Clinical improvement and endoplasmic reticulum stress genetic panel results were compared to the effectiveness of treatment.

## INTRODUCTION

Gastroesophageal reflux disease (GERD) and related disorders are important healthcare problems and have a significant impact on daily life.^1,2^ The imbalance between the defense mechanism of the esophagus and offensive factors of the stomach, such as acid, enzymes, and other digestive fluids, may trigger GERD. The prevalence of GERD is 13%-29% in America, 17% in Sweden, 10% in England, 10% in Spain, and 22.8% in Turkey. Although patients who suffer from GERD mostly complain of heartburn, regurgitation, and dysphagia, extraesophageal symptoms are rare. In general, GERD is diagnosed based on symptoms. Clinical history and presentation, response to proton pump inhibitors (PPIs), esophageal conventional 24-hour pH-meter or catheter-free pH-meter, and/or impedance studies are the other diagnostic options, but the absence of any “gold standard” test is the main problem in determining the diagnosis.^3,4^

Endoscopic findings of the upper gastrointestinal tract in patients with GERD are defined as nonerosive reflux disease (NERD), reflux esophagitis, and complications such as Barrett’s esophagus and strictures. Gastroesophageal reflux disease might be related to esophagitis, peptic esophageal ulcers, esophageal strictures, Barrett’s esophagus, and esophageal adenocarcinomas.^5^ Nonerosive reflux disease covers 3 different phenotypes: NERD, functional heartburn, and reflux hypersensitivity.^6^

A curative treatment for GERD is unavailable. Diet and lifestyle changes, such as avoiding tight clothes and not eating just before going to sleep, are treatment options. In 20%-25% of patients, PPIs are effective treatments, especially when used twice a day.^3^

The endoplasmic reticulum (ER) is an organelle that plays many essential roles in cells, including cleaning, protein synthesis, and cellular homeostasis. Disruption of ER functions by genetic or environmental factors causes misfolded or unfolded proteins to accumulate in the cell, which is defined as “endoplasmic reticulum stress” (ERS). Endoplasmic reticulum stress activates a homeostatic signaling pathway called the unfolded protein response (UPR).^7^ The UPR induces transcriptional and translational events to restore ER homeostasis. Chronic ERS and defects in UPR signaling may cause self-destruction of the cell and eventually apoptosis.^8^ The UPR is activated by signaling molecules (*IRE1*, *ATF6*, and *PERK*) located in the ER lumen or stress sensors in mammalian cells.^9,10^ Environmental factors that cause ERS, such as hypoxia, insufficient nutrition, and pH alterations, may be related to cancer.^11^ For example, one of the key components of the *UPR*, *XBP1*, is associated with esophageal cancer in mice, although the exact mechanism of this molecule in esophageal cancers remains unclear.^12^ But this study was performed with the mouse and not on human. Nonetheless, the structure of the human esophagus is completely different from that of the mouse esophagus, which is covered with keratinized epithelium. To date, no clear evidence of the effect of ERS marker expression on tumor prognosis or the outcomes of patients has been reported. The expression of ERS markers (*CHOP*, *XBP-1*, and *BIP*) is significantly decreased by revaprazan pretreatment compared to treatments with PPIs or gastroprotectant, accompanied by a significant reduction in the apoptotic index in cell cultures.^13^ Therefore, patients with gastric complaints are expected to show a reduction in the expression of stress markers at the molecular level following treatment.

In this study, we aimed to determine the role of ERS in the outcomes of patients suffering from gastroesophageal reflux disease and to evaluate temporal changes in the expression of ERS markers with treatment.

## MATERIALS AND METHODS

### Location of Study

The study was conducted at the Department of Gastroenterology and the Department of Medical Biology, Manisa Celal Bayar University, Manisa, between December 2018 and September 2019.

This study was performed in accordance with the Declaration of Helsinki, good clinical practice, and applicable regulatory requirements. Manisa Celal Bayar University Institutional Review Board approved this clinical trial on February 28, with number 2018/20.478.486. Each patient signed a consent form before any study-related procedure.

In our study, we made our diagnosis based on the clinic and symptoms. The pH meter diagnosis is not used for the first diagnosis in our clinic. Twenty-four subjects were recruited prospectively, of whom 15 had NERD (NERD, functional heartburn, or reflux hypersensitivity), and the remaining 9 had erosive reflux disease. Typical GERD symptoms were defined as at least 3 episodes of regurgitation and/or heartburn per week. Patients who were on continuous treatment with acid suppressants before upper gastrointestinal endoscopy; who had undergone upper gastrointestinal surgery such as gastrectomy, fundoplication, or distal esophagectomy; who had severe gastroparesis or esophageal varices and diabetes mellitus; and who had cases with infection criteria of C-reactive protein and procalcitonin elevation were excluded from the study. During upper gastrointestinal endoscopy, the distal 5 cm of the esophageal mucosal morphology at the squamocolumnar junction and gastric mucosa was visualized using conventional endoscopy with the narrow band imaging (NBI) system composed of video endoscopes (GIF-H260; Olympus), a video processor (Evis Lucera CV 260 SL; Olympus), and a lighting unit (Evis Lucerna CLV 260 SL; Olympus). Two experienced endoscopists (EK and TB) performed the endoscopic examinations. During standard white-light endoscopy and NBI examinations, erosions, mucosal breaks, and other complications were graded according to the Los Angeles classification. Two biopsies were collected 2 cm above the esophagogastric junction, 2 biopsies were collected from gastric antrum mucosa, and 2 biopsies were obtained from the gastric corpus mucosa with Olympus biopsy forceps. The biopsies were transferred in 10% formalin to the pathologist within 24 hours.

Simultaneously, 2 tubes of venous blood samples were drawn from each individual (1 tube was used to study the genetic markers and 1 tube was used to study the *CYP2C19* polymorphism). Symptoms were re-evaluated after 1 month of treatment. In addition, 1 tube of venous blood was drawn and stored after 1 month of treatment, as indicated in the protocol.

Whole blood was collected from patients into PAX gene Blood ribonucleic acid (RNA) tubes (Qiagen, Hilden, Germany). As stated in the user manual of the PAX gene Blood RNA tube, the tubes were gently mixed end-over-end 8 to 10 times instantly after blood collection. Each tube contained 2.5 mL of whole blood and 6.9 mL of additives.

Samples were frozen instantly using dry ice (a block of dry ice that has a surface temperature of −78.5°C) and stored at −80°C until RNA extraction.

### Ribonucleic Acid Extraction from Tissue and Blood

Total RNA was isolated using the RNeasy Mini Kit (Qiagen) according to the manufacturer’s protocol with slight modifications. For fresh tissue, samples (20-30 mg) from patients were disrupted with 600 μL of Buffer RLT and homogenized with a 7-mm diameter metal ball diameter in a Tissue Lyser II homogenizer (Qiagen) at 25 000 Hz for 5 minutes. The lysate was centrifuged at maximum speed, and the supernatant was treated according to the RNeasy Mini Kit protocol. For blood, RNA was extracted from PAX gene Blood RNA tubes, and routine quality control testing was performed. The PAX gene Blood RNA kit was used to extract RNA from the samples. The RNA was eluted with 75 mL of elution buffer and stored at −80°C. The quantity of total RNA was determined by measuring the absorbance at 260 nm (A260), and the RNA purity was determined by calculating the A260/A280 ratio using a spectrophotometer. The RNA quality was considered acceptable when the A260/A280 ratio was slightly higher than 2.0 and the A260/A230 ratio was slightly higher than 1.8. All steps were performed according to the manufacturer’s protocol. Complementary DNA (cDNA) synthesis was performed with an RT First Strand Kit (C-03) (SA Bioscience, Frederick, Md, USA). Eight microliters of the RNA sample were incubated with 2 μL of GE (5× gDNA Elimination Buffer) at 42°C for 5 minutes in a 0.2 mL polymerase chain reaction (PCR) tube. In another tube, a PCR cocktail (4 μL of RT Buffer 3 (5× BC3), 1 μL of P2 (Primer and External Control mix), 2 μL of RT Enzyme Mix 3 (RE3), and 3 μL of H_2_O were prepared and added to the RNA sample, followed by a 15-minute incubation at 42°C and a 5-minute incubation at 95°C. The cDNA samples were subsequently diluted as needed. All steps were performed according to the manufacturer’s protocol.

### Analysis of CYP2C19*2 and CYP2C19*3 Gene Polymorphisms

Gene polymorphisms in alleles were evaluated with commercially synthesized primary probes specific for the CYP2C19*2 and CYP2C19*3 alleles, and PPI metabolic states were analyzed (Table 1).

Genotyping of genomic DNA extracted from peripheral venous blood was performed according to the manufacturer’s instructions (TIB MOLBIOL GmbH, Berlin, Germany). Changes in both variants were detected simultaneously with the kit. Variants were detected using a LightCycler 1.5 real-time PCR device.

### Immunohistochemistry

Briefly, the tissue specimens were fixed with 10% neutral-buffered formalin for 24 hours before paraffin embedding and sectioning. Five-micrometer-thick sections were mounted on poly-d-lysine-coated slides. The tissue sections were rehydrated, incubated with citrate buffer at 95°C for 20 minutes, and treated with 0.3% H_2_O_2_ to quench endogenous peroxidase activity. After the rehydration periods, nonspecific antigen binding was prevented with a blocking reagent (IHC Select^®^ Blocking Reagent, Merck, Darmstadt, Germany, Millipore Cat≠20773) for 30 minutes at room temperature. According to the standard protocol, IHC for *ATF6* was performed with a rabbit polyclonal antibody against the human protein (1:200 dilution; Ab203119, Abcam, Cambridge, United Kingdom) at +4°C overnight. Then, sections were incubated with a 1:400 dilution of a biotin-conjugated secondary goat anti-rabbit antibody (Ab6720, Abcam) and streptavidin peroxidase for 90 minutes at room temperature. The sections were treated with diaminobenzidine tetrahydrochloride (DAB) for 5 minutes and counterstained with methyl green.

### Protein Extraction

Tissue specimens were homogenized in Radioimmunoprecipitation assay buffer (RIPA) buffer. After incubation in phenylmethanesulfonyl fluoride (Sigma, St. Louis, Missouri, USA, Cat. #D7626) and protease inhibitor cocktail (Sigma, Cat. #P8340) for 1 hour on dry ice, tissue extracts were centrifuged at 10,000 × *g* for 10 minutes at +4°C. Collected supernatants were centrifuged for the second time and aliquoted before the store in the deep freezer.

### Western Blotting

Protein concentrations were measured with a bicinchoninic acid (BCA) assay (Pierce BCA Protein Assay Kit, Thermo Scientific, Rockford, Ill, USA). Before loading on 4% stacking and 7.5% resolving polyacrylamide gels, protein concentrations were equalized to 20 µg/mL per well with RIPA buffer, denatured at 95°C for 4 minutes and 1:2 diluted with sample buffer. Sodium dodecyl sulfate-polyacrylamide gel electrophoresis was performed at 15 mA for 2 hours at room temperature in running buffer using the Mini-Protean III electrophoresis system (Bio-Rad Labs, Hercules, California, USA, Cat#165-3301). After separation, the gels were stained with 0.25% Coomassie brilliant blue. Gels were equilibrated with transfer buffer and sandwiched with polyvinylidene fluoride membranes (pore size 0.2 μm) before transferring at 90 mA overnight at +4°C using Mini Trans-Blot Electrophoretic Transfer Cell (Bio-Rad Labs, Cat#170-3930). Blotted proteins were visualized with 0.1% Ponceau-S (Sigma Cat#P3504), and membranes were blocked with nonfat dried milk (Sigma Cat#M7409) in Tris-buffered saline-Tween (TBST) buffer for 30 minutes at room temperature. After washes with TBST, membranes were incubated with previously optimized and 1:400 dilutions of the ATF6 MAb for 2 hours at room temperature and rinsed 3 times with TBST for 10 minutes. The membranes were probed with a goat anti-rabbit secondary antibody (Ab6720, Abcam) for 1 hour at room temperature and rinsed with TBST. A positive reaction was visualized with the DAB peroxidase substrate kit (Vector Lab, Newark, California, USA, Cat#SK-4100). After bands were obtained, the reaction was ceased with ultrapure water, and images of bands were acquired using an office scanner.

### Statistical Analysis

Statistical Package for the Social Sciences 15.0 (SPSS Inc.; Chicago, IL, USA) was used for the analysis of data. The parameters of blood and tissue samples before and after treatment were compared by the Mann–Whitney *U*-test. Comparison of gender, *Helicobacter pylori* (HP) in gastric antrum either/or corpus biopsy, esophagitis, *CYP2c19-2*, and usage of PPI were made by Wilcoxon signed-rank test. A *P*-value of <.05 was considered to be statistically significant.

## RESULTS

Twenty-four individuals (18 women and 6 men) with GERD symptoms were included in the study. The mean age was 42.3 ± 17.6 years for women and 34.66 ± 11.2 years for men. Nine patients were diagnosed with esophagitis based on the endoscopic examination. *H. pylori* was detected in 11 individuals. Pantoprazole, esomeprazole, rabeprazole, and lansoprazole preparations were used for treatment. Eight individuals carried CYP2C19*2 heterozygous mutations, and the remaining 16 individuals were wild-type for this allele. All of the cases were wild-type for the CYP2C19*3 allele. Before treatment, 15 (7 with and 8 without esophagitis) individuals experienced heartburn, and 19 (8 with and 11 without esophagitis) individuals experienced regurgitation. After treatment, 7 individuals (5 with and 2 without esophagitis) still experienced heartburn, and 7 individuals (5 with and 2 without esophagitis) had regurgitation (Table 1).

No significant differences in the expression of the panel of the *ATF-6*, *XBP-1*, *DDIT-3*, *DNAJC-10*, and *EIF-2-AK* genes were observed between tissue and blood samples before treatment (Table 2).

A significant decrease in the expression of the *ATF-6*, *XBP-1*, *DNAJC-9*, *EIF-2-AK*, and *NF-2L-2* mRNAs was observed in the blood after treatment (Table 3).

Significant decreases in the expression of the *ATF-6*, *XBP-1, EIF-2-AK, DNAJC-9*, and *NF-2L-2* mRNAs were observed in blood samples from female patients; in the expression of the A*TF-6, XBP-1, EIF-2-AK, DNAJC-9, ERN-1*, and *NF-2-L-2* mRNAs in blood samples from HP-positive patients; in the expression of the *ATF-6, XBP-1, DNAJC-9*, and *NF-2-L-2* mRNAs in patients without esophagitis in the endoscopic examination; and in the expression of the *ATF-6, XBP-1, EIF-2-AK, DNAJC-9,* and *NF-2-L-2* mRNAs in patients with wild-type *CYP2C19*.

In the comparison of PPIs, significant decreases in the expression of the *ATF-6, XBP-1,* and* DNAJC-9* mRNAs were detected in the blood of individuals after treatment (Table 4).

**Figure 1.**
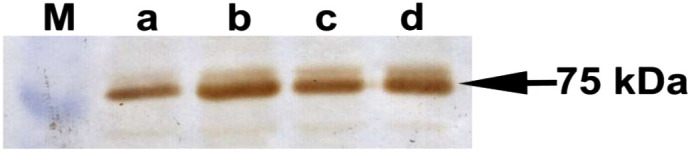
Western blot analysis of *ATF-6* in representing patients after the treatment. (A) HP-negative/esophagitis-negative/rabeprazole-positive male patient, (B) HP-positive/esophagitis-positive/pantoprazole-positive male patient, (C) HP-negative/esophagitis-negative/rabeprazole-positive female patient, and (D) HP-negative/esophagitis-positive/pantoprazole-positive male patient. HP, *Helicobacter pylori;* M, marker.

**Figure 2.**
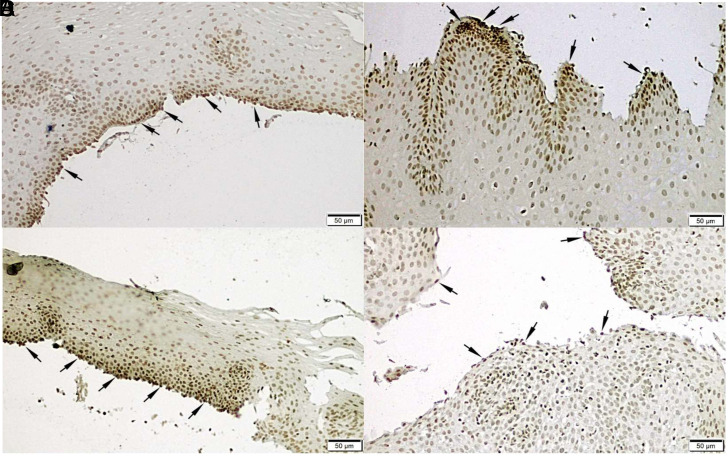
Immunohistochemical findings of tissue samples in WB. (A) HP-negative, esophagitis-negative, rabeprazole-positive male patient, (B) HP-positive, esophagitis-positive, pantoprazole-positive male patient, (C) HP-negative, esophagitis-negative, rabeprazole-positive female patient, and (D) HP-negative, esophagitis-positive, pantoprazole-positive male patient. HP, *Helicobacter pylori;* WB, Western blotting.

In addition to the RT-qPCR results esophagus, *ATF-6* expression in biopsies from randomly selected patients who received treatment was confirmed by performing IHC and western blotting (Figures 1 and 2).

## DISCUSSION

To the best of our knowledge, this study is the first to assess the expression of a panel of ERS-related genes in patients with GERD before and after treatment. The ER is an intracellular organelle that is responsible for maintaining cellular homeostasis, such as protein maturation and Ca^+2^ balance.^14^ Thus, when the intracellular balance is disrupted, the ER activates the UPR to protect intracellular homeostasis.^15^ Activation of *IRE-1* promotes the clipping of the mRNA encoding the transcription factor *XBP-1*. Then, *XBP-1* activates the gene expression of endoplasmic reticulum-associated protein degradation proteins, chaperones, and lipid synthesis enzymes responsible for the UPR. *ATF-6* is a transcription factor that is mainly located in the ER membrane. During unfolded protein accumulation, it is transported to the Golgi apparatus for activation. Then, it migrates to the nucleus to activate UPR-related protein expression. *PERK* is a protein kinase that suppresses *EIF2* activation by phosphorylation. Suppression of *EIF2* activity decreases protein synthesis and therefore the unfolded protein burden. *PERK* also promotes the UPR by inducing the activation of *ATF-4* transcription factors and inducing UPR-related protein expression.^15^

No differences in blood and tissue levels of *XBP-1*, *ATF-6*, and *EIF-2-AK* were observed before treatment; therefore, we speculated that we may be able to track the expression of these genes in blood. The evaluation of the expression of a panel of genes in blood revealed a significant decrease in the levels of *ATF-6, XBP-1, EIF-2-AK, DNAJC-9,* and* NF-2L-2* after treatment. Regarding the clinical symptoms with the statements of patients, a 63% decrease in regurgitation and 53% in heartburn were identified. Is a decrease in the expression of ERS markers an effective indicator of clinical symptoms? We answered this question by speculating that the treatment is efficient at both cellular and clinical levels. However, due to the decision of the ethics committee, second endoscopic examinations were not permitted for these patients. Studies enrolling a larger number of patients with repeated blood and endoscopic examinations are needed.

In our study, the expression of the *ATF-6, XBP-1, EIF-2-AK, DNAJC-9,* and* NF-2-L-2* mRNAs was significantly decreased in women after treatment. This finding might be related to hormonal differences, better treatment compliance (avoiding the foods that may cause GERD symptoms), or a large number of women in the study group.

A contradictory relationship between HP and GERD has been reported in population studies. However, the factors contributing to GERD must be considered to explain this finding, instead of the HP–GERD relationship alone.^16^ The exact relationship between HP and GERD is controversial; treatment of HP was reported to increase GERD symptoms in 2 meta-analyses.^17^ Bor et al^4^ reported no relationship between HP and GERD. On the other hand, in another meta-analysis, HP treatment decreased GERD symptoms.^18^ In our study, the expression of the *ATF-6, XBP-1, EIF-2-AK, DNAJC-9, ERN-1,* and* NF-2-L-2* mRNAs was significantly decreased after treatment in HP-positive patients. Acute HP infection is known to cause hypochlorhydria.^19,20^ We postulate that symptoms are rapidly suppressed in HP-positive individuals due to hypochlorhydria because the amount of acid is reduced. As a result, PPI treatment might have decreased ERS levels by protecting the esophageal mucosa from an acidic environment.

*ATF-6, XBP-1, DNAJC-9,* and* NF-2-L-2* levels were significantly decreased after treatment in patients without esophagitis in the endoscopic examination. The UPR activation results in a decrease in the rate of protein synthesis, an increase in protein folding and ER-related degradation, and eventually cell death.^8^ Proinflammatory signaling pathways that are activated during the UPR coordinate with nuclear factor kappa B (NF-κB) and activator protein-1 transcription factors.^19,20^ The ERS might induce NF-κB activation by decreasing *PERK*-related protein translation. This process causes a decrease in the level of the IκB protein and an increase in NF-κB levels. Both the *UPR* and inflammation are cellular response mechanisms that damage cells when dysregulated. In addition, *UPR* dysfunction plays a role in several autoimmune and inflammatory disorders, such as diabetes.^21^ The expression of ERS markers was significantly decreased in patients with esophagitis compared with patients without esophagitis. This difference might be attributed to the continuous and hard-to-suppress inflammation in these cases. If the results were evaluated after 5-6 months, likely no difference would be observed between the groups with and without esophagitis. The fact that we checked the results in a short period of 1 month might have affected the results.

*ATF-6, XBP-1 EIF-2-AK, DNAJC-9,* and* NF-2-L-2* levels were significantly decreased after treatment in patients with the wild-type *CYP2C19* polymorphism. The PPI metabolism and pharmacokinetics are regulated by cytochrome P450 enzymes (particularly S-phenytoin-4-hydroxylase), which are encoded by *CYP2C19*.^22^ A Turkish population study of patients with dyspepsia revealed that CYP2C19*2 and CYP2C19*3 genotypes were detected in 13% and 1% of patients, respectively.^23^ The heterozygous mutant CYP2C19*2 G/A allele was detected in 16.6% of patients in our study. Significant decreases in *ATF-6, XBP-1 EIF-2-AK, DNAJC-9,* and* NF-2-L-2* levels after treatment were attributed to the stronger effect of acid suppression by PPIs in patients with the wild-type genotype.

The PPIs, which were used for treatment in this study, were randomly selected. The PPIs were more effective at decreasing the levels of *ATF-6, XBP-1,* and* DNAJC-9*.

In this study, the therapeutic response was evaluated in conjunction with symptoms after 1 month of PPI therapy. Due to the small number of patients, PPI drugs were not categorized to differentiate the response. We speculated that the inhibition of acid erosion by PPI in the esophageal mucosa might suppress the expression of the ERS-related gene panel. The main weakness of this study is that the esophageal mucosa was not evaluated with second control endoscopic examinations. However, due to the decision of the ethics committee, second endoscopic examinations of the patients were not permitted. Therefore, we were unable to show the second endoscopic and macroscopic results.

We propose that further studies may address the questions listed below. (i) Can ERS be used to assess the effectiveness of treatment in patients with GERD? (ii) Can ERS be used as a therapeutic target? (iii) Can ERS be used as a screen for complications in patients with GERD? We suggest that future studies should include pre- and post-therapeutic esophageal epithelial tissues with objective healing measurements.

In conclusion, the clinical improvement and results from the ERS-related gene panel correlated well with our study.

## Figures and Tables

**Table 1.** Demographic Clinical and Laboratory Findings of the Cases Included in the Study

						GERD Symptom		CYP2c19 G (Wild Type -Healthy) G/A Heterozygous Mutation	
								Patient Number	Gender	Age	Endoscopic Finding	HP	PPI	Heartburn BT/AT	Regurgitation BT/AT	CYP2c19*2.	CYP2c19*3
1	F	47	Normal endoscopy	Positive	Pantoprazole	−/−	+/+	G allele	G allele
2	F	46	Esophagitis LA grade B, gastritis, bulbit	Negative	Rabeprazole	+/−	+/−	G allele	G allele
3	F	41	Normal endoscopy	Positive	Esomeprazole	−/−	+/−	G allele	G allele
4	F	37	Pangastritis	Positive	Rabeprazole	−/−	+/−	G allele	G allele
5	F	21	Normal endoscopy	Negative	Pantoprazole	−/−	+/−	G allele	G allele
6	M	34	Normal endoscopy	Negative	Rabeprazole	−/−	+/−	G allele	G allele
7	F	32	Normal endoscopy	Negative	Pantoprazole	+/+	−/−	G/A	G allele
8	F	40	Esophagitis LA grade B	Negative	Esomeprazole	+/+	+/−	G/A	G allele
9	F	47	Esophagitis LA grade B	Negative	Esomeprazole	+/−	+/+	G/A	G allele
10	M	51	Esophagitis LA grade B	Positive	Esomeprazole	+/+	−/−	G allele	G allele
11	M	37	Esophagitis LA grade B	Positive	Pantoprazole	+/−	+/−	G allele	G allele
12	F	42	Normal endoscopy	Positive	Esomeprazole	+/−	+/−	G allele	G allele
13	F	59	Esophagitis LA grade B, pangastritis	Negative	Esomeprazole	−/−	+/+	G allele	G allele
14	F	29	Normal endoscopy	Negative	Rabeprazole	+/−	+/−	G/A	G allele
15	F	37	Bulbus ulcus	Positive	Pantoprazole	+/−	−/−	G/A	G allele
16	F	70	Gastritis	Negative	Pantoprazole	+/−	+/−	G allele	G allele
17	M	24	Pangastritis	Negative	Rabeprazole	−/−	+/−	G allele	G allele
18	M	29	Esophagitis LA grade B, pangastritis	Negative	Pantoprazole	+/+	+/+	G/A	G allele
19	F	49	Normal endoscopy	Negative	Esomeprazole	+/−	−/−	G allele	G allele
20	F	63	Esophagitis LA grade B, bulbus ulcus	Positive	Rabeprazole	+/+	+/+	G allele	G allele
21	F	19	Normal endoscopy	Positive	Rabeprazole	−/−	+/−	G allele	G allele
22	F	24	Normal endoscopy	Negative	Rabeprazole	+/+	−/−	G/A	G allele
23	F	60	Normal endoscopy	Positive	Lansoprazole	−/−	+/+	G allele	G allele
24	M	33	Esophagitis LA grade B	Positive	Pantoprazole	+/+	+/+	G/A	G allele

AT, after treatment; BT, before treatment; GERD, gastroesophageal reflux disease; *H. pylori*, *Helicobacter pylori*; PPI, proton pump inhibitor.

**Table 2.** Comparison of Blood and Tissue Values Before Treatment

	Blood (Mean ± SD)	Tissue (Mean ± SD)	*P*
ATF-4	6.93 ± 2.04	5.21 ± 1.40	**.001**
ATF-6	9.42 ± 1.36	9.58 ± 1.34	.675
XBP-1	8.72 ± 2.88	7.46 ± 2.71	.220
DDIT-3	10.44 ± 1.12	9.85 ± 1.19	.087
DNAJB-9	11.64 ± 2.23	8.89 ± 1.55	**<.001**
ERN-1	9.27 ± 1.12	11.16 ± 1.96	**<.001**
DNAJC-10	9.38 ± 1.95	8.36 ± 1.50	.048
EIF-2-AK-3	11.98 ± 1.70	8.57 ± 2.03	.053
NF-2L-2	8.57 ± 2.03	4.70 ± 1.88	**<.001**

P < .05 values that indicated in bold are significant.

**Table 3.** Comparison of Blood Values Before and After Treatment

	BT (Mean ± SD)	AT (Mean ± SD)	*P*
ATF-4	6.93 ± 2.04	6.97 ± 3.64	.963
ATF-6	9.42 ± 1.36	8.40 ± 0.99	**.003**
XBP-1	8.72 ± 2.88	6.82 ± 1.53	**.022**
DDIT-3	10.44 ± 1.12	10.37 ± 1.39	.889
DNAJB-9	11.64 ± 2.23	10.13 ± 1.09	**.005**
ERN-1	9.27 ± 1.12	8.95 ± 1.26	.407
DNAJC-10	9.38 ± 1.95	9.06 ± 2.86	.666
EIF-2-AK-3	11.98 ± 1.70	10.52 ± 1.88	**.025**
NF-2L-2	8.57 ± 2.03	7.60 ± 1.06	**.040**

AT, after treatment; BT, before treatment.

**Table 4.** Comparison of Gender, *H. pylori*, Esophagitis, *CYP2c19-2*, and PPI Before and After Treatment

		Gender		*H. pylori*		Esophagitis		CYP2c19-2		PPI *P*
		F	M	+	−	+	−	G	G/A	
Atf-4	BT	7.10 ± 2.30	6.43 ± 0.90	6.85 ± 2.31	6.99 ± 1.87	7.30 ± 2.21	6.71 ± 1.98	7.29 ± 2.38	6.21 ± 0.81	6.93 ± 2.10
	AT	7.25 ± 4.12	6.11 ± 1.42	6.98 ± 4.05	6.96 ± 3.47	6.70 ± 4.24	7.13 ± 3.38	7.62 ± 4.11	7.62 ± 4.11	6.96 ± 2.76
	*P*	.586	.753	.424	.861	.594	.609	.605	.674	.829
Atf-6	BT	9.56 ± 1.50	8.99 ± 0.75	10.02 ± 1.76	8.91 ± 0.61	8.96 ± 0.65	9.69 ± 1.61	9.63 ± 1.56	6.21 ± 0.81	9.42 ± 1.13
	AT	8.25 ± 1.10	8.86 ± 0.17	8.44 ± 0.80	8.36 ± 1.16	8.25 ± 1.31	8.49 ± 0.78	8.53 ± 0.63	5.67 ± 2.10	8.40 ± 0.84
	*P*	**.001**	.600	**.004**	.117	.260	**.002**	**.001**	.208	**.029**
Xbp-1	BT	9.02 ± 2.99	7.83 ± 2.49	8.73 ± 2.42	8.71 ± 3.31	9.86 ± 3.98	8.04 ± 1.78	9.11 ± 2.62	7.94 ± 3.37	8.72 ± 2.82
	AT	6.71 ± 1.37	7.16 ± 2.05	6.47 ± 1.14	7.12 ± 1.78	6.47 ± 2.23	7.04 ± 0.93	6.72 ± 1.15	7.02 ± 2.18	6.82 ± 1.33
	*P*	**.010**	.686	**.016**	.505	.110	**.056**	**.005**	.726	**.026**
DDIT 3	BT	10.48 ± 0.99	10.31 ± 1.56	10.76 ± 0.94	10.16 ± 1.23	10.48 ± 1.54	10.41 ± 0.85	10.61 ± 0.93	10.08 ± 1.44	10.43 ± 1.04
	AT	10.32 ± 1.41	10.52 ± 1.41	10.00 ± 1.08	10.69 ± 1.58	9.99 ± 1.75	10.60 ± 1.12	10.51 ± 1.13	10.10 ± 1.87	10.37 ± 1.21
	*P*	1.000	.917	.142	.173	.859	.650	.897	.575	.580
DNAJB-9	BT	11.87 ± 2.32	10.95 ± 1.94	12.63 ± 2.71	10.80 ± 1.32	10.99 ± 1.60	12.03 ± 2.50	12.24 ± 2.36	10.45 ± 1.40	11.64 ± 2.26
	AT	10.06 ± 1.20	10.32 ± 0.69	9.97 ± 0.92	10.26 ± 1.24	9.76 ± 1.32	10.35 ± 0.91	10.11 ± 0.83	10.16 ± 1.55	9.12 ± 0.97
	*P*	**.008**	.463	**.010**	.422	.214	**.027**	**.002**	.889	**.012**
ERN 1	BT	9.45 ± 1.02	8.70 ± 1.31	9.58 ± 0.92	8.99 ± 1.24	9.21 ± 1.31	9.30 ± 1.03	9.56 ± 0.97	9.56 ± 0.97	9.26 ± 0.93
	AT	8.89 ± 1.30	9.08 ± 1.23	8.67 ± 0.89	9.18 ± 1.50	8.51 ± 1.68	9.20 ± 0.89	8.99 ± 0.95	8.99 ± 0.95	8.94 ± 0.88
	*P*	.157	.753	**.033**	.600	.314	.570	.079	.779	.098
DNAJC-10	BT	9.55 ± 2.15	8.86 ± 1.20	9.94 ± 2.69	8.92 ± 0.87	9.20 ± 1.14	9.50 ± 2.34	9.65 ± 2.25	8.87 ± 1.11	9.38 ± 1.65
	AT	9.08 ± 3.18	8.99 ± 1.74	8.51 ± 1.34	9.52 ± 3.70	8.21 ± 2.00	9.57 ± 3.22	9.17 ± 3.22	8.86 ± 2.13	9.06 ± 2.37
	*P*	.102	.753	.062	.972	.314	.532	.109	1.000	.174
EIF-2-AK-3	BT	12.14 ± 1.41	11.50 ± 2.49	12.42 ± 2.03	11.61 ± 1.33	12.09 ± 2.30	11.91 ± 1.30	12.16 ± 1.76	11.63 ± 1.63	11.97 ± 1.45
	AT	10.83 ± 1.67	9.60 ± 2.30	10.23 ± 1.91	10.77 ± 1.89	9.29 ± 2.17	11.26 ± 1.24	10.63 ± 1.88	10.32 ± 1.99	10.52 ± 1.65
	*P*	**.058**	.463	**.010**	.807	**.051**	.307	**.056**	.401	.059
NF-2-L-2	BT	8.78 ± 2.23	7.96 ± 1.21	9.40 ± 2.65	7.87 ± 0.93	7.76 ± 1.06	9.05 ± 2.34	9.07 ± 2.26	7.58 ± 0.96	8.57 ± 1.85
	AT	7.51 ± 1.13	7.85 ± 0.83	7.54 ± 0.73	7.64 ± 1.30	7.27 ± 1.43	7.79 ± 0.75	7.77 ± 0.76	7.24 ± 1.49	7.59 ± 0.89
	*P*	**.035**	.463	**.008**	.861	.594	**.015**	**.011**	.674	.429

AT, after treatment; BT, before treatment; *H. pylori*, *Helicobacter pylori*; PPI, proton pump inhibitor.

## References

[1] El-Serag HB Sweet S Winchester CC Dent J . Update on the epidemiology of gastroesophageal reflux disease: a systematic review. Gut. 2014;63(6):871 880. (10.1136/gutjnl-2012-304269) 23853213 PMC4046948

[2] Tack J Becher A Mulligan C Johnson DA . Systematic review: the burden of disruptive gastro-oesophageal reflux disease on health-related quality of life. Aliment Pharmacol Ther. 2012;35(11):1257 1266. (10.1111/j.1365-2036.2012.05086.x) 22486579

[3] Unal Ng B Gastroözofagial Reflü S . Hastalığı. Turk Klin Gastroenterohepatoloji Özel Derg. 2011;4:9 25.

[4] Bor S Kitapcioglu G Kasap E . Prevalence of gastroesophageal reflux disease in a country with a high occurrence of *Helicobacter pylori* . World J Gastroenterol. 2017;23(3):525 532. (10.3748/wjg.v23.i3.525) 28210089 PMC5291858

[5] Turcotte S Duranceau A . Gastroesophageal reflux and cancer. Thorac Surg Clin. 2005;15(3):341 352. (10.1016/j.thorsurg.2005.03.003) 16104125

[6] de Bortoli N Ottonello A Zerbib F Sifrim D Gyawali CP Savarino E . Between GERD and NERD: the relevance of weakly acidic reflux. Ann N Y Acad Sci. 2016;1380:218 229. 27472432 10.1111/nyas.13169

[7] Travers KJ Patil CK Wodicka L Lockhart DJ Weisman JS Walter P . Functional and genomic analyses reveal an essential coordination between the unfolded protein response and ER-associated degradation. Cell. 2000;101:249 258. 10847680 10.1016/s0092-8674(00)80835-1

[8] Hsiao JR Chang KC Chen CW , et al. Endoplasmic reticulum stress triggers XBP-1-mediated up-regulation of an EBV oncoprotein in nasopharyngeal carcinoma. Cancer Res. 2009;69(10):4461 4467. (10.1158/0008-5472.CAN-09-0277) 19435892

[9] Hetz C . The unfolded protein response: controlling cell fate decisions under ER stress and beyond. Nat Rev Mol Cell Biol. 2012;13(2):89 102. (10.1038/nrm3270) 22251901

[10] Schröder M Kaufman RJ . The mammalian unfolded protein response. Annu Rev Biochem. 2005;74:739 789. (10.1146/annurev.biochem.73.011303.074134) 15952902

[11] Kim KM Yu TK Chu HH , et al. Expression of ER stress and autophagy-related molecules in human non-small cell lung cancer and premalignant lesions. Int J Cancer. 2012;131:362 370. 10.1002/ijc.26463 21953091

[12] Rosekrans SL Heijmans J Büller NV , et al. ER stress induces epithelial differentiation in the mouse oesophagus. Gut. 2015;64(2):195 202. (10.1136/gutjnl-2013-306347) 24789843

[13] Kim JH Kim EH Ock C , et al. Mitigating endoplasmic reticulum stress with revaprazan ameliorates stress-related mucosal disease. J Gastroenterol Hepatol. 2012;27(1):120 129. (10.1111/j.1440-1746.2011.06838.x) 21722181

[14] Ellgaard L Helenius A . Quality control in the endoplasmic reticulum. Nat Rev Mol Cell Biol. 2003;4(3):181 191. (10.1038/nrm1052) 12612637

[15] Cooper GM Hausman RE . Protein classification and transport. Endosplasmic reticulum, Golgi apparatus and lysosomes (Turkish version). Cell Mol Approach. 2016:10:416p.

[16] Scida S Russo M Miraglia C , et al. Relationship between *Helicobacter pylori* infection and GERD. Acta Biomed. 2018;89(8-S):40 43. (10.23750/abm.v89i8-S.7918) PMC6502218 30561416

[17] Mazzoleni F Mazzoleni LE de Magalhães Francesconi CF , et al. Potential roles of* Helicobacter pylori* treatment, body mass index and waist circumference in the causation of erosive esophagitis: a randomized clinical trial (HEROES-GERD) . Int J Obes. 2020;44:147 158. (https://doi.org/10.1038/s41366-019-0391-3 ) 10.1038/s41366-019-0391-3 31197249

[18] Saad AM Choudhary A Bechtold ML . Effect of *Helicobacter pylori* treatment on gastroesophageal reflux disease (GERD): meta-analysis of randomized controlled trials. Scand J Gastroenterol. 2012;47(2):129 135. (10.3109/00365521.2011.648955) 22229305

[19] Smolka AJ Schubert ML . *Helicobacter pylori* induced changes in gastric acid secretion and upper gastrointestinal disease. Curr Top Microbiol Immunol. 2017;400:227 252. (10.1007/978-3-319-50520-6_10) 28124156

[20] Charles EH Craig B Giovanni S Richard MP Steffen B Adam JS . *Helicobacter pylori* virulence factors affecting gastric proton pump expression and acid secretion. Am J Physiol Gastrointest Liver Physiol. 2015;309:193 201. 10.1152/ajpgi.00099.2015 PMC4525105 26045613

[21] Hotamisligil GS . Endoplasmic reticulum stress and the inflammatory basis of metabolic disease. Cell. 2010;140(6):900 917. (10.1016/j.cell.2010.02.034) 20303879 PMC2887297

[22] Ormeci A Emrence Z Baran B , et al. Can *Helicobacter pylori* be eradicated with high-dose proton pump inhibitör in extensive metabolizers with CYP2C19 genotypic polymorphism? Eur Rev Med Pharmacol Sci. 2016;20:1795 1797. 27212172

[23] Çelebi A . The prevalence of CYP2C19 mutations in Turkish patients with dyspepsia and influence on *H. pylori* eradication therapy. Turk J Gastroenterol. 2012;23(6):805 806. (10.4318/tjg.2012.0405) 23864462

